# *Notes from the Field:* Multiple Cruise Ship Outbreaks of Norovirus Associated with Frozen Fruits and Berries — United States, 2019

**DOI:** 10.15585/mmwr.mm6916a3

**Published:** 2020-04-24

**Authors:** Jared R. Rispens, Amy Freeland, Beth Wittry, Adam Kramer, Leslie Barclay, Jan Vinjé, Aimee Treffiletti, Keisha Houston

**Affiliations:** ^1^Epidemic Intelligence Service, CDC; ^2^Division of Environmental Health Science and Practice, National Center for Environmental Health, CDC; ^3^Division of Viral Diseases, National Center for Immunizations and Respiratory Diseases, CDC.

From July to September 2019, cruise line X experienced sudden, unexplained outbreaks (>3% of the passenger population) of acute gastroenteritis (AGE) among passengers on 10 cruise ships sailing in Europe. The rapid onset of vomiting and diarrhea followed by recovery within 24 hours were consistent with norovirus infection. Investigations by the cruise line throughout the summer yielded no clear source of the outbreaks even after extensive food testing. On September 18, 2019, CDC’s Vessel Sanitation Program (VSP) was notified of an outbreak of AGE on cruise ship A of cruise line X, sailing into U.S. jurisdiction (defined as passenger vessels carrying ≥13 passengers sailing to the United States from a foreign port) from Germany to New York City (*1*). By the end of the 19-day voyage on September 23, a total of 117 of 2,046 (5.7%) passengers and eight of 610 (1.3%) crew members met the case definition for AGE (three or more loose stools within a 24-hour period or more than normal for the patient, or vomiting plus one other sign or symptom including fever, diarrhea, bloody stool, myalgia, abdominal cramps, or headache). Four stool specimens were collected and tested for norovirus at CDC’s National Calicivirus Laboratory; three tested positive for norovirus by quantitative reverse transcription–polymerase chain reaction (RT-PCR). No outbreak source was determined after a field investigation by a VSP team on September 22.

The following month, on October 7, CDC’s VSP was notified of two more outbreaks in U.S. jurisdiction. The first outbreak occurred on another ship (ship B) of cruise line X sailing to and from New York City along the eastern seaboard and affected 85 (3.9%) of 2,166 passengers and 10 (1.6%) of 612 crew members; the second outbreak occurred on ship A sailing from Montreal to New York City and affected 83 (3.7%) of 2,251 passengers and 10 (1.6%) of 610 crew members. VSP again conducted outbreak investigations on October 12 (ship B) and October 13 (ship A). Five stool specimens from ship B and two from ship A were collected for laboratory testing. During the field investigations, cruise line X’s public health officials reported to VSP that after reviewing food questionnaires completed by ill passengers on ship B, nearly 80% of completed questionnaires implicated a smoothie made from frozen fruits and berries. Because of the epidemiologic link and because berries have been implicated in past outbreaks (*2*,*3*), CDC requested assistance from the Food and Drug Administration (FDA) to collect frozen fruit and berry items from ship B for norovirus testing. Food item lot numbers from ship B matched those from the same frozen fruit and berry items on ship A.

Overall, nine of 11 stool samples from the three outbreak voyages on ships A and B tested positive for norovirus by quantitative RT-PCR at CDC; these included three of four from ship A’s first voyage, four of five from ship B, and two of two from ship A’s second voyage. The samples were typed as GII.2[P16]. FDA tested 16 frozen fruit and berry items, and three items tested positive for norovirus: raspberries (norovirus genogroup II), tropical fruit cocktail (norovirus genogroup I), and berry mix (norovirus genogroup I). Norovirus sequences from the stool samples and from raspberries were 97.5% similar. After removal of the fruit items, no further outbreaks were reported on cruise line X.

Upon further review of food provisioning, cruise line X determined that its food vendor had purchased several containers (nearly 22,000 pounds) of frozen raspberries of the same lot from a supplier in China beginning the end of June 2019. These raspberries had been supplied to the entire fleet of cruise line X. Both the epidemiologic and laboratory data implicated the raspberries as the cause of the outbreaks. As a result of these findings, on November 11, the World Health Organization issued a recall notice* for frozen raspberries traced back to China. This investigation highlights the importance of AGE surveillance at sea to prevent transmission of AGE illness through U.S. ports and to identify contaminated foods at sea that had not yet been implicated on land.

## Footnotes

* https://webgate.ec.europa.eu/rasff-window/portal/?event=notificationDetail&NOTIF_REFERENCE=2019.3843.
